# Discrepancies Exist between Exercise Prescription and Dose in Elite Women’s Basketball Pre-Season

**DOI:** 10.3390/sports8050070

**Published:** 2020-05-19

**Authors:** Craig Staunton, Daniel Wundersitz, Brett Gordon, Michael Kingsley

**Affiliations:** 1 Williamstown Football Club, Williamstown, Victoria 3106, Australia; 2 Holsworth Research Initiative, La Trobe Rural Health School, La Trobe University, Bendigo 3552, Australia; d.wundersitz@latrobe.edu.au (D.W.); b.gordon@latrobe.edu.au (B.G.); michael.kingsley@auckland.ac.nz (M.K.); 3 Department of Exercise Sciences, Faculty of Science, University of Auckland, Auckland 1023, New Zealand

**Keywords:** accelerometers, training load, monitoring, intensity

## Abstract

This study assessed the influence of exercise prescription on the objectively measured exercise dose in basketball. Intensity (RPE) and volume (sRPE) were prescribed by a professional coach on a drill-by-drill basis during pre-season training for nine elite basketball players. Training drills were classified by prescribed intensity (easy-moderate, moderate-hard, hard–very hard, and very hard-maximal) and type (warm-up, skill-development, offensive- and defensive-technical/tactical, or match-simulation). Exercise intensity was objectively quantified using accelerometry-derived average net force (AvF_Net_) and time spent in accelerometry-derived relative intensity zones. The volume of exercise (exercise dose) was objectively quantified using accumulated impulse (AvF_Net_ × duration). Relationships between prescribed volume and exercise dose were explored by correlations between sRPE and drill-by-drill accumulation of sRPE (dRPE) with impulse. Very hard-maximal drill intensity was greater than hard-very hard (*p =* 0.011), but not moderate-hard (*p* = 0.945). Very hard-maximal drills included the most time performing Supra-maximal intensity (>100% $\dot{V}$O_2_R) efforts (*p* < 0.001), suggesting that intensity prescription was based upon the amount of high-intensity exercise. Correlations between impulse with sRPE and dRPE were moderate (*r* = 0.401, *p* = 0.197) and very-large (*r* = 0.807, *p =* 0.002), respectively, demonstrating that the coach misinterpreted the accumulative effect of drill volume over an entire training session. Overall, a mismatch existed between exercise prescription and exercise dose. Objective monitoring might assist coaches to improve precision of exercise prescription.

## 1. Introduction

Differences between coaches’ and players’ perceptions of effort are widely acknowledged in a range of sports [1,2,3,4,5,6,7]. These differences suggest that discrepancies might exist between the volume of prescribed exercise and the exercise dose that players receive. Discrepancies between exercise prescription and exercise dose could cause maladaptation to the training program and either under- or over-training [7]. Furthermore, the mismatch between prescribed exercise volume and exercise dose has been suggested to be even more pronounced in team sports such as basketball because management of group exercise can be challenging [2].

Previous research has identified that different intensities and/or different training drills can influence the mismatch between perceptions of effort [4,6,7]. For example, volleyball coaches underestimated the perceived exertion of players, particularly during high-intensity physical conditioning drills [6]. However, the volume of technical–tactical drills prescribed with moderate-intensity closely matched the received exercise dose [6]. A limitation common to these studies is the use of subjective ratings of perceived exertion (RPE) to determine the exercise dose received by players. RPE includes components of emotion, which can influence the score reported during physical effort [8]. Objective measurement is required to remove the emotional component of perceived exertion in order to isolate the actual exercise dose received by players. Consequently, objectively determined exercise dose is required to explain the potential mediating influences of prescribed intensity and drill type on the physical demand placed on players.

Typically, time-motion analyses have been used to objectively monitor exercise dose in basketball [9,10,11]. Measuring movement patterns through time-motion analyses (e.g., speed of movement and distances covered) has utility for quantifying the volume and intensity of continuous activities. However, recent research has demonstrated that this practice underestimates the intensity of some sport-specific movements, such as jumping, shuffling and changes of direction [12], which occur frequently during basketball training [13] and competition [9,10,11].

More recently, accelerometers have been proposed as a measurement tool for monitoring the exercise dose in basketball [13]. Accelerometry-derived average net force (AvF_Net_) is a measurement of exercise intensity. Previous research has confirmed the construct validity of AvF_Net_ during basketball-specific movement patterns [12]. Additionally, AvF_Net_ has proven utility to quantify relative exercise intensity in basketball match-play [14] and training sessions [15].

Only one previous study has assessed the association between exercise prescription and objectively measured exercise dose [3]. A moderate positive correlation was reported to exist between exercise prescription and accelerometry-derived exercise dose in elite junior soccer for an entire training session. However, this study did not assess the mismatch between coaches’ exercise prescription and the exercise dose received by players’ for individual training drills. Therefore, it remains unknown whether the mismatch between exercise prescription and exercise dose is consistent for the intensity of activity or type of drill performed. Additionally, the characteristic movement patterns performed in basketball training differ from those commonly undertaken during soccer training. Therefore, this study aimed to identify if discrepancies exist between the volume and intensity of exercise prescribed by a coach to the exercise dose actually completed by players during basketball pre-season training drills.

## 2. Materials and Methods

### 2.1. Participants

Nine professional female basketball players (age: 26 ± 3 years; stature: 180 ± 6 cm; body mass: 79 ± 7 kg; $\dot{V}$O_2peak_ 49 ± 6 mL·kg^−1^·min^−1^) participated in this study. All players were from the Women’s National Basketball League (WNBL), which is the peak level of competition in Australia. Written informed consent was obtained from all participants prior to undertaking this study. The La Trobe University Human Research Ethics Committee (ref: UHEC 15-088) provided approval for this study and the study followed the 2013 Code of Ethics of the World Medical Association (Helsinki Declaration).

### 2.2. Study Design

Players completed a preliminary data collection session followed by a 6-week mesocycle of pre-season basketball training. The preliminary data collection session included basic anthropometry and a modified Yo-Yo intermittent recovery test (level 1; modified Yo-Yo-IR1) to provide a controlled calibration to which the intensity of training drills were compared. Stature (Portable Stadiometer; Holtain, UK) and body mass (SE762 Mechanical Scale; Seca, Denmark) were measured according to guidelines [16]. Following this, all players completed the modified Yo-Yo-IR1 as previously described [14,15]. In short, seven slower stages ranging from 3 to 9 km·h^−1^ were included to represent low intensity movement, such as walking, that typically occur during competition [9,11]. The first 3 km·h^−1^ stage was repeated twice and then each subsequent stage increased the speed by 1 km·h^−1^ before starting the standard Yo-Yo-IR1, which was performed until exhaustion.

An upper-back mounted three-dimensional accelerometer sensors sampling at 100 Hz with a dynamic range of ± 8g (Link; ActiGraph, Pensacola, FL, USA) was worn by all players throughout the study period, as previously described [12,14]. The accelerometer has previously been shown to be reliable [17,18,19]. Further, breath-by-breath oxygen consumption (Oxycon Mobile, Jaeger, Germany) was recorded during the modified Yo-Yo-IR1 to establish accelerometry and $\dot{V}$O_2_ relationships between individual participants.

Prior to all basketball training sessions, the head coach prescribed intensity and duration for each drill and for the overall session, for all nine players individually. A 10-point rating of perceived exertion (RPE) scale was used to prescribe exercise intensity [20]. The head coach had over 10 years’ experience coaching elite level basketball and more than one year of experience using RPE. All training drills were categorised into different types as follows: warm-up (WU), skill-development drills (SD), offensive technical/tactical drills (OD), defensive technical/tactical drills (DD), and match-simulation drills (MS). Players typically completed three on-court training sessions per week, with each lasting approximately 90–120 min. Briefly, WU drills consisted of dynamic movements, such as skipping, shuffling, lunging, and squatting, along the basketball court with the intention of gradually increasing intensity to physically prepare players for the upcoming training session. SD drills consisted of drills related to practicing shooting and dribbling. For example, a typical SD drill was a shoot and rebound drill performed in small teams. OD and DD drills consisted of semi-match simulation drills where there were uneven numbers of offensive and defensive players (e.g., 3v2 with focus on certain offensive and defensive tactical strategies). MS drills were competitive 5v5 match-simulation drills with fouls and time-outs called by the head coach.

### 2.3. Data Analysis

Training drills prescribed by the coach were classified to be easy-moderate (RPE 0–3), moderate-hard (RPE 3–5), hard-very hard (RPE > 5–7), and very hard-maximal (RPE > 7–10). Volume of exercise prescription was quantified from session-RPE (sRPE) as the product of the total session prescribed intensity (RPE) and the total session prescribed duration (minutes) [20,21]. Additionally, volume of exercise prescription was also quantified for all separate drills using drill RPE (dRPE), which was calculated from the product of the prescribed intensity (RPE) and prescribed duration (minutes) for separate drills and accumulated for the total session.

Manufacturer’s software was used to download accelerometer data (ActiLife version 12; ActiGraph, Pensacola, FL, USA). Average net force, derived from accelerometer data, was used to quantify exercise intensity as previously described [12,14,15]. Specifically, a dual-pass, fourth order Butterworth filter was used to filter acceleration data. To remove gravity from the accelerometer signal a high pass cut-off frequency of 0.1 Hz was chosen [22,23] and to remove noise from the signal a low pass cut-off frequency of 15 Hz was selected [24,25]. To determine instantaneous net force (F_Net_), the instantaneous acceleration vector was multiplied by body mass and the average F_Net_ (AvF_Net_) was calculated in epochs of 1-s from user-selected periods using customised software (LabVIEW 2016; National Instruments, Austin, TX, USA). Interpolated $\dot{V}$O_2_ was also analysed using the customised software and included in the output as described previously [15]. Accumulated impulse (Impulse; N·s), the numerical integral of AvF_Net_ and exercise duration, was used to calculate the exercise dose for training drills and the entire session.

To determine resting $\dot{V}$O_2_, the five minutes of seated rest before beginning the modified Yo-Yo-IR1 was used. To synchronise $\dot{V}$O_2_ and accelerometer data, the acceleration signal during the initial shuttle was reconciled with the start of $\dot{V}$O_2_ recording. Then for the start of every stage, the acceleration signal was designated from movement commencement (i.e., the time-point when resultant acceleration values began to rise from stationary values) to the successful end of the entire 40-m stage. To determine Peak $\dot{V}$O_2_, the highest 5-s average $\dot{V}$O_2_ value achieved during the last successfully completed stage was used. To calculate $\dot{V}$O_2_ reserve ($\dot{V}$O_2_R), relative to each individual so that it would be representative of their relative maximum $\dot{V}$O_2_ above rest, resting $\dot{V}$O_2_ values were subtracted from the calculated peak $\dot{V}$O_2_ values. Correlations and best-fit linear relationships between AvF_Net_ and average $\dot{V}$O_2_R for each stage were generated for all participants. A very strong relationship was found for all participants (r^2^ = 0.87–0.99).

Accelerometer data were recorded from the start of the warm-up to the end of the last drill/ cool-down. This included all training activities, time-outs, and stoppages. The accelerometer signal of the entire training session was graphically displayed using the aforementioned customised software, which also permitted sections to be made. These sections, which split each individual training drill, were reconciled with a recorded timestamp of when each training drill commenced and ended, thus permitting the accelerometer signal of each individual training drill to be analysed.

Training drill predicted $\dot{V}$O_2_R was determined from AvF_Net_ in one second epochs using the same linear relationship developed for each participant from the Yo-Yo-IR1. Seven intensity zones were used to categorise relative exercise intensity as defined by the American College of Sports Medicine [26]. These were <20% $\dot{V}$O_2_R (sedentary); 20%–<30% $\dot{V}$O_2_R (very light); 30% –< 40% $\dot{V}$O_2_R (light); 40% –< 60% $\dot{V}$O_2_R (moderate); 60% –< 90% $\dot{V}$O_2_R (vigorous); 90% –< 100% $\dot{V}$O_2_R (maximal); and ≥100% $\dot{V}$O_2_R (supra-maximal). The proportion of time and total time in every intensity zone was calculated for all participants during all training drills. All participants’ outcome measures were calculated and separated by drill type (WU, SD, OD, DD, or MS) or by coach intensity prescription (easy-moderate, moderate-hard, hard-very hard, or very hard-maximal).

### 2.4. Statistical Analysis

The program SPSS Statistics for Windows (Version 26.0; IBM Corporation, Armonk, NY, USA) was used for all statistical analyses and statistical significance set at *p* < 0.05. The assumption of normality was assessed using Shapiro–Wilk tests and all data were normally distributed (*p* > 0.05). As a result, mean ± standard error of the mean (SEM) was used. The effects of exercise prescription on objectively measured exercise intensity were assessed using two separate repeated measures two-way ANOVAs (within factors: exercise intensity—% time in exercise intensity zones; exercise prescription—coach intensity prescription or drill type). Significant interaction effects (exercise intensity × exercise prescription) were explored using simple main effects analysis with Bonferroni correction. Additionally, separate one-way ANOVAs were used to assess the effect of drill type on the average exercise intensity during the drill (prescribed (RPE) or objectively measured (AvF_Net_)). Significant main effects were followed by pairwise comparisons with Bonferroni correction. Greenhouse–Geisser correction was applied if Mauchly’s test of sphericity was violated.

Differences in the drill-by-drill contributions to overall exercise volume between prescribed exercise volume and received exercise dose were assessed using repeated measures mixed-model ANOVAs (within factor: drill type; between factor: exercise volume (dRPE or Impulse)). Significant interaction effects were followed up with simple main effect analyses with Bonferroni correction. Bivariate Pearson’s product moment correlations were used to assess relationships between prescription of exercise volume and exercise dose. The relative strength of these relationships were classified according to previously described thresholds: moderate: 0.3-0.5; large: 0.5-0.7; and very large: 0.7–0.9 [27].

## 3. Results

Across the six week training mesocycle, mean session duration was 107 ± 5 min, consisting of 9.5 ± 0.4 separate training drills per session. There were 61 drills prescribed as moderate-hard, 70 prescribed as hard-very hard, and 51 prescribed as very hard-maximal. There were no drills prescribed in the easy-moderate category. 

**Figure 1:**
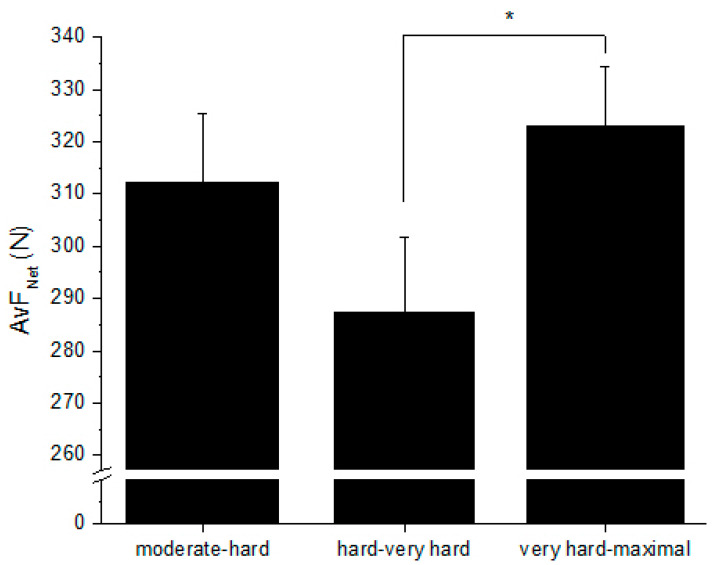
Average net force (AvF_Net_) for moderate-hard (RPE 3–5), hard-very hard (RPE > 5–7), and very hard-maximal (RPE > 7–10) intensity prescription. Mean ± SEM. * Difference in AvF_Net_ between hard-very hard and very hard-maximal prescribed intensities (*p* = 0.011).

The AvF_Net_ for all prescribed intensity categories are presented in Figure 1. Exercise intensity received by players differed across the prescribed intensities (main effect: *F*(2, 16) = 8.260, *p* = 0.003), where AvF_Net_ was greater for very hard-maximal compared to hard-very hard (323 ± 12 N vs. 287 ± 14 N; *p =* 0.011), but not different to moderate-hard (312 ± 13 N; *p* = 0.945).

The proportional time spent in the exercise intensity zones for all prescribed intensity categories are displayed in Table 1. The coach’s prescription of exercise intensity influenced the proportional time spent in the relative exercise intensity zones (exercise prescription × exercise intensity interaction: *F*(12, 96) = 14.403, *p* < 0.001). Drills prescribed to be moderate-hard involved a lower proportion of sedentary behaviour (36% ± 4%), compared to drills prescribed to be hard-very hard (43% ± 4%; *p <* 0.001) and very hard-maximal (43% ± 3%; *p =* 0.012). On the other hand, drills prescribed to moderate-hard involved a greater proportion of light- (12% ± 2%) and moderate-intensity (11% ± 0%) activity compared to hard-very hard (10% ± 2%, 8% ± 0%; *p* ≤ 0.001) and very hard-maximal drills (10% ± 2%, 7% ± 0%; *p* ≤ 0.007). As expected, drills prescribed to be very hard-maximal involved more supra-maximal activity (7% ± 0%), compared to moderate-hard (3% ± 0%; *p =* 0.013) and hard-very hard (4% ± 3%; *p* = 0.011).

The prescribed RPE, AvF_Net_ and proportional time spent in each exercise intensity zone for all drill types are presented in Table 2. Prescribed and received exercise intensity were influenced by the drill type (main effect: *F*(4, 32) ≥ 18.026, *p* < 0.001). Prescribed RPE was lowest for WU drills compared to all other drill types (*p* < 0.001) and greatest for DD and MS (*p* ≤ 0.031). AvF_Net_ was greater for WU when compared to all other drills except SD (*p* ≤ 0.035). Drill type influenced the proportional time spent in the relative exercise intensity zones (drill type × exercise intensity interaction: *F*(24,192) = 24.625, *p* < 0.001). WU drills involved a lower proportion of sedentary behaviour compared to all other drill types (*p* ≤ 0.001), but more light (*p* ≤ 0.002) and moderate-intensity (*p* < 0.001) activity. Additionally, WU drills had a greater proportion of vigorous-intensity activity compared to all drills except SD (*p* ≤ 0.002). Drill type had no influence on the proportion of supra-maximal intensity activity (*p* ≥ 0.064).

**Figure 2:**
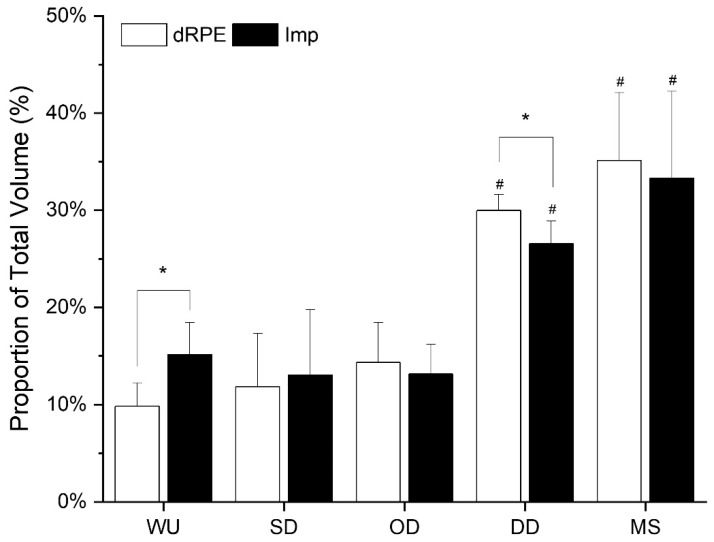
Proportion of average dRPE and impulse for each drill type. Mean ± SEM. * Difference between conditions dRPE vs. impulse (*p <* 0.001). ^#^ DD and MS different to all other drills types (*p* ≤ 0.032). WU = warm-up, SD = skill-development drills, OD = offensive technical/tactical drills, DD = defensive technical/tactical drills, MS = match-simulation drills. dRPE = drill rating of perceived exertion, Impulse = accumulated impulse.

The proportional time that each drill type contributed to the total prescribed exercise volume and received exercise dose are displayed in Figure 2. The pattern of drill-by-drill contribution to overall exercise volume was different for prescribed exercise volume compared to received exercise dose (drill type × volume type interaction: *F*(4, 32) = 33.245, *p* < 0.001). Prescription of exercise volume for WU drills was lower than the exercise dose received (*p* < 0.001). The prescribed exercise volume for DD was higher than the exercise dose received (*p* < 0.001). For both impulse and dRPE, DD and MS both contributed the greatest proportions of total volume compared to WU, SD and OD (*p* ≤ 0.032).

There was a moderate and insignificant positive correlation between average impulse and sRPE (*r* = 0.401, *p* = 0.197). Conversely, the correlation between impulse and dRPE was very large and significant (r = 0.807, *p* = 0.002).

## 4. Discussion

The findings of this study confirmed a mismatch between exercise prescription and objectively determined exercise dose during pre-season training in elite basketball players. Drills prescribed at a moderate-hard intensity resulted in higher exercise intensities than prescribed. As a result, the volume of exercise prescription for warm-up drills was lower than the exercise dose received. The prescription of exercise intensity was proportional to the amount of time players spent in the supra-maximal intensity zone, suggesting that the coach might base intensity prescription on the amount of high-intensity exercise completed by players. Although the coach’s prescription of volume was similar to the exercise dose completed by players when accumulated on a drill-by-drill basis, the coach misinterpreted the accumulation of drill volume over an entire training session when providing an assessment of the overall session intensity.

The received exercise intensity for moderate-hard drills was similar to that of very hard-maximal drills. This finding indicates that there was a mismatch between the exercise intensity prescribed by the coach and the exercise intensity received by players, particularly when drills were intended to be less intense. This finding is consistent with previous research, which has identified similar coach-player mismatches in perceptions of effort [1,2,3,4,5,6,7]. The present study confirmed that coach–athlete mismatches in perceived training dose extend to objectively determined physical demand during basketball specific training. Underestimation of the exercise dose over a prolonged period could lead to maladaptive training, inadequate recovery, and an increased risk of injury and/or overtraining [7,28].

The amount of time that players spent in the supra-maximal intensity zone increased with intensity of exercise prescription. This finding suggests that the coach might prescribe intensity of exercise based upon the amount of high-intensity efforts required in the intended training drill. For example, WU drills were prescribed with the lowest RPE and objectively had the least proportional time spent in the supra-maximal intensity zone compared to all other drill types. This result suggests that coaches might be able to accurately predict the amount of time players will perform high-intensity efforts and base their prescription of intensity upon this prediction. The ability of coaches to predict the amount of time players perform high-intensity efforts is useful because previous research has identified associations between the amount of high-intensity effort and increased risk of soft-tissue injuries [29,30]. However, solely basing prescription of intensity upon the intended amount supra-maximal intensity exercise might not truly reflect the average intensity experienced over the duration of a drill. For example, the coach underestimated the intensity of moderate-hard drills, likely because the amount of sedentary activity was not considered or well predicted and the proportion of moderate- and vigorous-intensity activity was underestimated.

Despite the ability to accurately predict the amount of high-intensity activity, the coach tended to underestimate total exercise volume. The mismatch between dRPE and impulse suggests that the coach underestimated the contribution of warm-up drills to the overall exercise dose received. Providing objective feedback to coaches about the exercise intensity and dose received by players might allow coaches to calibrate their exercise prescriptions in order to avoid maladaptive training. 

The predisposition for the coach to misinterpret total exercise volume, was further evidenced by the moderate and insignificant relationship between prescribed exercise volume (sRPE) and the received exercise dose (impulse). However, the correlation between dRPE and impulse was very large and significant. This result is congruent with data from elite junior tennis, where coaches’ underestimated athlete session RPE but not drill RPE [4]. Taken together, it seems likely that coaches misjudge the accumulating effect of drill volume over an entire training session. Objective monitoring of the exercise dose received during training and match-play can assist coaches to optimise adaptation; however, if this is not possible, coaches can more accurately estimate the exercise dose of sessions by accumulating subjective drill volume and not estimating the overall session intensity.

## 5. Conclusions

There was a mismatch between exercise prescription and exercise dose during pre-season training in an elite women’s basketball team. Exercise dose received was greater than the exercise volume prescribed for some drills. The accumulative effect of drill volume over an entire training session was underestimated. These mismatches between exercise prescription and exercise dose could lead to maladaptation of the training program and place athletes at an increased risk of injury and overtraining. In order to optimise adaptation, coaches should objectively monitor the exercise dose received by players during training and match-play.

## Figures and Tables

**Table 1:** Proportional time spent in relative exercise intensity zone for moderate-hard (RPE 3–5), hard-very hard (RPE > 5–7), and very hard-maximal (RPE > 7–10) intensity prescription.

	Exercise Prescription		
Intensity Zone	Moderate-hard	Hard-very Hard	Very Hard-maximal
Sedentary (%)	36 ± 4 *	43 ± 4	43 ± 3
Very light (%)	21 ± 1 ^#^	20 ± 2	20 ± 1
Light (%)	12 ± 2 *	10 ± 2	10 ± 2
Moderate (%)	11 ± 1 *	8 ± 1 ^#^	7 ± 1
Vigorous (%)	13 ± 1 ^	11 ± 1	11 ± 1
Maximal (%)	3 ± 1	3 ± 0	3 ± 0
Supra-maximal (%)	3 ± 1	4 ± 1	7 ± 2 *

Mean ± SEM. * Different to all other exercise prescription intensities (*p* < 0.05); ^ different to hard-very hard (*p* < 0.05); and ^#^ different to very hard-maximal (*p* < 0.05). Sedentary (<20% $\dot{V}$O_2_R); Very light (20%–<30% $\dot{V}$O_2_R); Light (30%–<40% $\dot{V}$O_2_R); Moderate (40%–<60% $\dot{V}$O_2_R); Vigorous (60%–<90% $\dot{V}$O_2_R); Maximal (90%–<100% $\dot{V}$O_2_R); and Supra-maximal (≥100% $\dot{V}$O_2_R; [26]).

**Table 2:** Duration, rating of perceived exertion (RPE), average net force (AvF_Net_), and proportional time spent in each exercise intensity zone for each drill type.

			Drill Type		
Intensity	WU	SD	OD	DD	MS
Time (min)	11.8 ± 0.4	9.1 ± 0.3	7.5 ± 0.2	8.8 ± 0.1	12.3 ± 0.2
RPE (AU)	4.8 ± 0.1 *	6.5 ± 0.2 ^	6.0 ± 0.1 ^	7.4 ± 0.0	7.4 ± 0.0
AvF_Net_ (N)	365 ± 17 *	342 ± 15	269 ± 13	306 ± 12	299 ± 16
Sedentary (%)	23 ± 4 *	37 ± 5 ^#^	48 ± 5	43 ± 4 ^#^	45 ± 4
Very light (%)	23 ± 1	20 ± 2	19 ± 2	22 ± 2	19 ± 2
Light (%)	16 ± 2 *	11 ± 2	9 ± 2	10 ± 2	9 ± 1
Moderate (%)	17 ± 1 *	9 ± 1 ^#^^	7 ± 0	7 ± 1	8 ± 1
Vigorous (%)	16 ± 1 *	14 ± 1 ^#^	9 ± 1	10 ± 1	11 ± 1 ^
Maximal (%)	3 ± 1	4 ± 1 ^#^	3 ± 0	3 ± 0	3 ± 0
Supra-maximal (%)	3 ± 1	5 ± 2	5 ± 2	6 ± 1	5 ± 2

Mean ± SEM. * Different to all other drill types (*p* < 0.05); ^#^ different to OD (*p* ≤ 0.05); and ^ different to DD (*p* ≤ 0.05). WU = warm-up, SD = skill-development drills, OD = offensive technical/tactical drills, DD = defensive technical/tactical drills, MS = match-simulation drills. Sedentary (<20% $\dot{V}$O_2_R); Very light (20%–<30% $\dot{V}$O_2_R); Light (30%–<40% $\dot{V}$O_2_R); Moderate (40%–<60% $\dot{V}$O_2_R); Vigorous (60%–<90% $\dot{V}$O_2_R); Maximal (90%–<100% $\dot{V}$O_2_R); and Supra-maximal (≥100% $\dot{V}$O_2_R; [26]).
